# Effects of Glycine Supplementation on Mitochondrial Function and Protein Degradation in Skeletal Muscle of Old Mice

**DOI:** 10.1093/geroni/igab046.3592

**Published:** 2021-12-17

**Authors:** Giulia Lizzo, Kamila Muller, Jonathan Thevenet, Stefan Christen, Kim Zarse, Michael Ristow, Nabil Bosco, Philipp Gut

**Affiliations:** 1 Nestlé Research, Lausanne, Vaud, Switzerland; 2 ETHZ, Zurich, Zurich, Switzerland; 3 Cell Biology, Nestlé Institute of Health Sciences, Nestlé Research, Lausanne, Vaud, Switzerland

## Abstract

Glycine is the simplest amino acid and it has a pivotal role in different metabolic processes, such as being a building block of glutathione, collagen and purine bases, or taking part in methylation reactions, detoxication and ammonia metabolism. Although considered for many years a non-essential amino acid, glycine levels are decreased in certain conditions, as the endogenous synthesis cannot fulfill the needs required to sustain all the cellular processes in which glycine is involved. Here we describe that glycine levels are significantly lower in skeletal muscle of aged zebrafish and mice and in plasma of humans compared to young subjects. We therefore fed healthy old mice for 6 weeks with a glycine-supplemented diet and observed a significant restoration of glycine levels in skeletal muscle and liver towards young mouse levels. Moreover, old mice showed decreased mitochondrial function in glycolytic and oxidative fibers, and a significant increase in oxygen consumption was observed in glycolytic fibers after glycine supplementation. The improvement of mitochondrial function is not associated to an increased mitochondrial biogenesis or an increased antioxidant capacity, but glycine supplementation increases both total GSH and GSSG levels, suggestive of a pro-oxidant environment. Overall, glycine supplementation induced an increase in the cross-sectional area of fibers. Finally, we carried out RNA-Seq study to decipher the impact of higher glycine intake. Our results suggest that age-associated glycine deficiency plays an important role in atrophy of muscle, especially in glycolytic fibers, and is reversible with a dietary supplementation.

